# Extended-time of Noninvasive Positive Pressure Ventilation Improves Tissue Perfusion after Coronary Artery Bypass Surgery: a Randomized Clinical Trial

**DOI:** 10.21470/1678-9741-2017-0232

**Published:** 2018

**Authors:** Mara L. S. Nasrala, Douglas W. Bolzan, Yumi G. Lage, Fabiana S. Prado, Ross Arena, Paulo R. L. Lima, Gibran Feguri, Ageo M. C. Silva, Natasha O. Marcondi, Nelson Hossne, Solange Guizilini, Walter J. Gomes

**Affiliations:** 1 Cardiology and Cardiovascular Surgery Disciplines, Escola Paulista de Medicina, Universidade Federal de São Paulo (EPM-UNIFESP), São Paulo, SP, Brazil.; 2 Physical Therapy Department, Hospital Santa Rosa, Cuiabá, MT, Brazil.; 3 Physical Therapy Department, Hospital São Mateus, Cuiabá, MT, Brazil.; 4 Department of Physical Therapy, College of Applied Health Sciences, University of Illinois Chicago, Chicago, IL, USA.; 5 Public Health Department, Universidade Federal do Mato Grosso (UFMT), Cuiabá, MT, Brazil.; 6 Department of the Human Movement Sciences, Universidade Federal de São Paulo (UNIFESP), São Paulo, SP, Brazil.

**Keywords:** Coronary Artery Bypass, Lactic Acid/Blood, Lung/Physiology, Forced Expiratory Volume, Positive-Pressure Respiration.

## Abstract

**Objective:**

To compare the effects of extended- *versus* short-time noninvasive positive pressure ventilation on pulmonary function, tissue perfusion, and clinical outcomes in the early postoperative period following coronary artery bypass surgery in patients with preserved left ventricular function.

**Methods:**

Patients were randomized into two groups according to noninvasive positive pressure ventilation intensity: short-time noninvasive positive pressure ventilation n=20 (S-NPPV) and extended-time noninvasive positive pressure ventilation n=21 (E-NPPV). S-NPPV was applied for 60 minutes during immediate postoperative period and 10 minutes, twice daily, from postoperative days 1-5. E-NPPV was performed for at least six hours during immediate postoperative period and 60 minutes, twice daily, from postoperative days 1-5. As a primary outcome, tissue perfusion was determined by central venous oxygen saturation and blood lactate level measured after anesthetic induction, immediately after extubation and following noninvasive positive pressure ventilation protocols. As a secondary outcome, pulmonary function tests were performed preoperatively and in the postoperative days 1, 3, and 5; clinical outcomes were recorded.

**Results:**

Significant drop in blood lactate levels and an improvement in central venous oxygen saturation values in the E-NPPV group were observed when compared with S-NPPV group after study protocol (*P*<0.01). The E-NPPV group presented higher preservation of postoperative pulmonary function as well as lower incidence of respiratory events and shorter postoperative hospital stay (*P*<0.05).

**Conclusion:**

Prophylactic E-NPPV administered in the early postoperative period of coronary artery bypass surgery resulted in greater improvements in tissue perfusion, pulmonary function and clinical outcomes than S-NPPV, in patients with preserved left ventricular function.

**Trial Registration:**

Brazilian Registry of Clinical trial - RBR7sqj78 - http://www.ensaiosclinicos.gov.br

**Table:** 

Abbreviations, acronyms & symbols				
BIPAP	= Bilevel positive airway pressure		IPO	= Immediate postoperative period
BMI	= Body mass index		LITA	= Left internal thoracic artery
CABG	= Coronary artery bypass surgery		LVEF	= Left ventricular ejection fraction
CPB	= Cardiopulmonary bypass		NPPV	= Noninvasive positive pressure ventilation
CVP	= Central venous pressure		PEEP	= Positive end-expiratory pressure
E-NPPV	= Extended-time noninvasive positive pressure ventilation		POD	= Postoperative day
FEV_1_	= Forced expiratory volume in 1 second		ScvO_2_	= Central venous oxygen saturation
FiO_2_	= Inspired oxygen fraction		S-NPPV	= Short-time noninvasive positive pressure ventilation
FVC	= Forced vital capacity		SpO_2_	= Arterial oxygen saturation
ICU	= Intensive care unit			

## INTRODUCTION

Elevated blood lactate level and low central venous oxygen saturation (ScvO_2_) have been independently associated with an increased risk of complications and longer postoperative hospital stay following cardiac surgery, contributing to increased morbidity and mortality after coronary artery bypass surgery (CABG)^[^^1^^-^^3^^]^. In addition, several studies have shown that postoperative pulmonary dysfunction following CABG is inevitable, which could increase the occurrence of respiratory complications and delay recovery^[^^4^^-^^6^^]^.

Noninvasive positive pressure ventilation (NPPV) has been used to accelerate the recovery of pulmonary function as well as to prevent and treat postoperative pulmonary complications. Previous evidence indicates a significant drop in blood lactate concentration 60 minutes following NPPV^[^^7^^]^.

To date, no study has addressed the influence of early use of NPPV on key measures and clinical outcomes following cardiac surgery. In this context, the aim of the current study was to compare the effects of extended- *versus* short-time prophylactic NPPV, applied in the early postoperative period following CABG, on pulmonary function parameters, tissue perfusion determined by ScvO_2_, blood lactate level, and clinical outcomes. We hypothesized that NPPV would provide numerous beneficial effects and that the extended-time mode would be superior to short-time NPPV.

## METHODS

This randomized controlled trial was conducted between June 2013 and May 2014, at Santa Rosa and São Mateus Hospitals, Brazil. All ethical aspects were respected, with approval of the institutions' Clinical Ethical Research Committees. All subjects were informed about the study and they have signed a written consent form prior to the enrollment.

### Patients

Patients undergoing elective first-time on-pump CABG were prospectively included in the current study. Inclusion criteria were: both genders and 18 years of age or older. Inability to perform spirometry, hemodynamic instability, left ventricular ejection fraction less than 45%, emergency surgery, chronic or acute pulmonary disease, intraoperative death, renal failure (creatinine > 1.3 mg/dL), anatomical abnormalities interfering with NPPV mask fit, obesity [*i.e*., body mass index (BMI) > 30], steroid treatment, and uncooperative state served as primary exclusion criteria.

The patients were prospectively randomized into two groups: short-time NPPV (S-NPPV; n=20) and extended-time NPPV (E-NPPV; n=21). A random sequence was performed through a software on "random.org" and allocation secrecy was kept by numbered, sealed, opaque envelopes.

### Surgical Procedure

All patients received the same anesthetic regimen during CABG. Anesthesia was induced in a routine manner with etomidate and midazolam and maintained with fentanyl and sevoflurane (0.5% to 1%). Mechanical ventilation was started with volume-controlled ventilation at the following settings: 1) tidal volume at 8 ml/kg of predicted body weight; 2) positive end-expiratory pressure (PEEP) at 0 cmH_2_O; 3) inspiration/expiration ratio at 1:2; 4) inspired oxygen fraction (FiO_2_) set to keep oxygen saturation above 90%; and 5) respiratory rate adjusted to achieve a PaCO_2_ between 35 and 45 mmHg. During the operation, mean arterial pressure, central venous pressure (CVP), arterial blood gas, temperature, urine output, electrocardiography, and heart rate were continuously monitored.

Operation was performed through a median sternotomy, using the left internal thoracic artery (LITA) graft, which was harvested according to the skeletonized technique and complemented with additional saphenous vein grafts. Meticulous care was routinely taken to preserve the pleura integrity during LITA harvesting. In all patients, before chest closure, in the presence of incidental left pleura opening, a soft tubular PVC drain was inserted and exteriorized at the subxiphoid region and positioned in the left costophrenic sinus. In all subjects, a mediastinal drain was also placed via a subxiphoid entry.

Cardiopulmonary bypass (CPB) was established with ascending aorta cannulation and single cannula venous drainage, after systemic heparinization to keep the activated coagulation time above 480 seconds. Myocardial protection was achieved using intermittent hypothermic antegrade blood cardioplegia, associated with systemic mild hypothermia (34ºC).

### Postoperative Management

All patients were transferred to the intensive care unit (ICU) and ventilated on volume-controlled ventilation using the following parameters: 12-14 breaths/minute with a FiO_2_ level set to maintain arterial oxygen saturation (SpO_2_) above 90%; inspiratory/expiratory ratio of 1:2; PEEP of 5 cmH_2_O; and pressure support to maintain a tidal volume of 8 ml/kg of predicted body weight. Extubation was performed when patients were hemodynamically stable and alert to maintain self-ventilation and good blood gas values. All patients received the same analgesic protocol (100 mg of tramadol chlorhydrate, 4 times a day) administered until postoperative day (POD) 5. Patients also underwent daily physical therapy sessions until discharge. Chest tubes were routinely removed on POD2 and patients had daily chest X-ray.

### Study Design

Following extubation, all E-NPPV patients received NPPV with bilevel positive airway pressure (BIPAP) for at least six hours in the immediate postoperative period (IPO) and 60 minutes, twice a day, from POD1 to POD5. S-NPPV patients received NPPV with BIPAP administered for 1 hour in the IPO period and 10 minutes, twice a day, from POD1 to POD5, according to the ICU routine. In both groups, during NPPV application, patients were in semi-recumbent position, with the head of the bed elevated at 45°. BiPAP(r) Synchrony(r) equipment (Respironics) was used with an adjustable face mask with the following parameters: inspiratory positive airway pressure sufficient to ensure a tidal volume of 8 ml/kg; and PEEP of 10 cmH_2_O with FiO_2_ adjusted to maintain SpO_2_>90%.

### End Points

As a primary outcome, tissue perfusion was determined by ScvO_2_ and blood lactate level. As a secondary outcome, pulmonary function and clinical outcomes were assessed.

### Tissue Perfusion

For tissue perfusion analysis, blood samples directly drawn from the CVP catheter were analyzed to assess the ScvO_2_ and, simultaneously, blood samples collected from the arterial catheter were used to evaluate blood lactate levels. Low ScvO_2_ was defined as <65% and hyperlactatemia was defined as a blood lactate level >3 mmol/l. Blood samples were collected in three moments at the IPO: intraoperative period (after anesthesia induction and invasive mechanical ventilation), immediately after extubation (spontaneous ventilation), and immediately after the NPPV protocol.

### Pulmonary Function

Forced vital capacity (FVC) and forced expiratory volume in 1 second (FEV_1_) were evaluated at the bedside on the day before the operation and repeated on POD1, 3, and 5 (after NPPV protocols) by the same respiratory physiotherapist, using a portable spirometer (Spirobank G, MIR, Rome, Italy), according to the American Thoracic Society standards^[^^8^^]^.

### Clinical Outcomes

Length of mechanical ventilation and duration of postoperative hospital stay were recorded for all patients. A radiologist who was blinded to subject group allocation evaluated chest roentgenograms taken preoperatively and through POD1 to POD5. Respiratory events were also evaluated (atelectasis, pleural effusions, and pneumonia). Pleural effusion was considered relevant when exceeding the phreno-costal angle and fluid drainage was monitored hourly^[^^4^^]^. Atelectasis was acknowledged when a clear atelectasis radiological shadow exceeded 15 mm in width^[^^4^^]^; linear atelectasis was disregarded in this study. Pneumonia was defined by the presence on chest radiographs with new or persistent pulmonary infiltrates not otherwise explained, in combination with at least two of the following criteria: body temperature of >38°C; leukocytosis (>10,000 cells/mm^3^); and purulent respiratory secretions^[^^9^^]^. Assessors blinded to group allocation documented the incidence of respiratory events.

### Statistical Analysis

Data are reported as mean ± standard deviation. Based on previous studies^[^^4^^]^, sample size calculation was based on FVC at POD1, considering a significance level of 5% and 80% power to detect a difference between groups of at least a 400 ml decrease compared to the preoperative period. This assumption suggested a sample of 40 patients, resulting in a total of 60 patients recruited to account for patients not completing the study^[^^10^^]^. Initially, the Kolgomorov-Smirnov test was applied to determine the distribution of variables. When variables were compared between groups, we used the unpaired Student's t-test; and Mann-Whitney test was used when deemed necessary. For intragroup analysis, we used the paired Student's t-test; and ANOVA was used for repeated measures, as appropriate. For categorical data, the Pearson's chi-square test was performed. The Pearson correlation coefficient was used to evaluate associations.

In this study, clinically relevant threshold values of 3 mmol/l for blood lactate level and 65% for the ScvO_2_ were used. Trend analysis of these variables was performed using simple linear regression models. The construction of scatter plots of the variables showed in all cases that a linear evaluation could be assumed, which supported the use of this model. Simple linear regression models were adjusted for each group. As a measure of precision of these models, we used the coefficient of determination (r^2^), later transformed into Pearson's correlation coefficient. Statistical analysis was performed by computerized statistical program (SPSS13.0, Chicago, IL, USA) software. For all statistical tests, a *P*-value <0.05 defined statistical significance.

## RESULTS

**Fig. 1:**
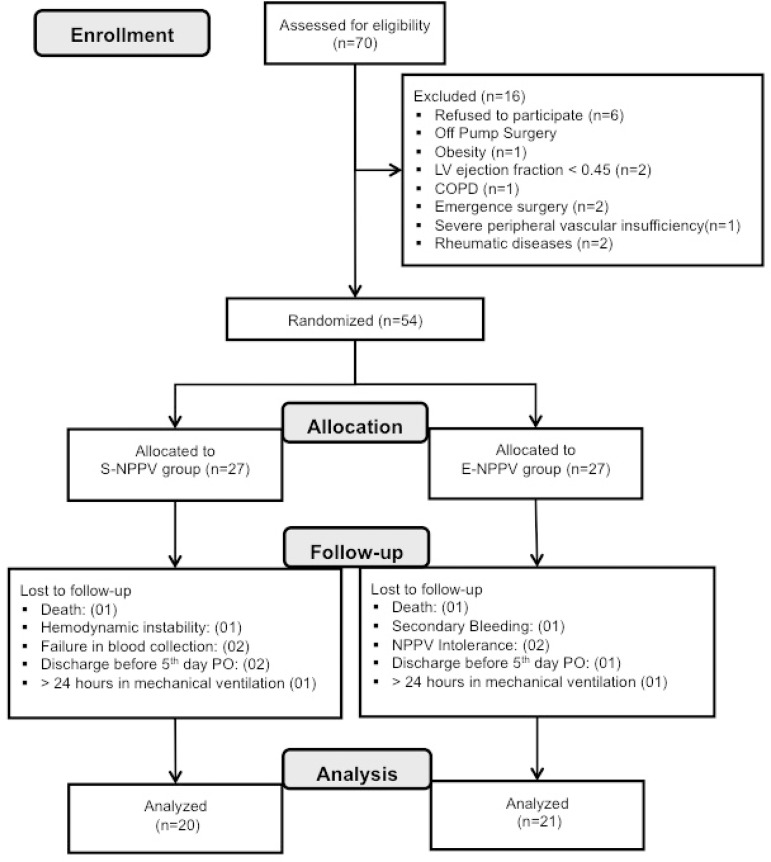
Flowchart of consecutive CABG patients enrolled in the study. CABG=coronary artery bypass surgery; COPD=chronic obstructive pulmonary disease; E-NPPV=extended-time noninvasive positive pressure ventilation; LV=left ventricular; PO=postoperative; S-NPPV=short-time noninvasive positive pressure ventilation

During the study period, 70 patients were assessed for eligibility. From that sample, 16 were excluded, 54 were randomized, and 41 were, in fact, analyzed (Figure 1).

The groups were homogeneous; pre- and intraoperative patients' characteristics are summarized in Table 1. Following extubation, patients in the E-NPPV group received prophylactic NPPV for 6.21±0.44 hours.

**Table 1:** Pre- and intraoperative patients' characteristics.

Variables	S-NPPV Group (n=20)	E-NPPV Group (n=21)	*P* value
Age (years)	59.7±11.4	58.6±7.3	0.36
Men % (n)	85.0 (17)	76.2 (16)	0.17
BMI (kg/m^2^)	27.4±5.0	27.7±4.0	0.80
LVEF (%)	61.2±14.6	63.0±11.3	0.66
CPB time (min)	71.1±19.9	70.5±27.0	0.71
Aortic cross-clamp time (min)	55.2±15.2	53.2±20.5	0.52
Operative time (h)	4.2±1.3	3.7±0.9	0.51
Grafts per patient (n)	2.9±1.0	2.6±0.9	0.26
Pulmonary function			
FVC (l)	3.3±0.9	3.1±0.8	0.40
% predicted	94.21±18.2	91.72±15.9	0.35
FEV_1_ (l)	2.8±0.7	2.9±0.7	0.42
% predicted	88.7±18.0	90.1±16.1	0.29
Pleurotomy % (n)	15.0 (3)	9.5 (2)	0.30

Data are shown as mean ± standard deviation. BMI=body mass index; CPB=cardiopulmonary bypass; E-NPPV=extended-time noninvasive positive pressure ventilation; FEV1=forced expiratory volume in 1 second; FVC=forced vital capacity; LVEF=left ventricular ejection fraction; S-NPPV=short-time noninvasive positive pressure ventilation

A significant increase in blood lactate levels and a drop of ScvO_2_ were observed in both groups after extubation in comparison with pre-anesthetic values (*P*<0.05). A strong negative correlation (r=-0.84) was observed between blood lactate levels and ScvO_2_ after trend analysis by linear regression, with all patients breathing spontaneously (before NPPV application) during the IPO period (*P*<0.001).

**Fig. 2:**
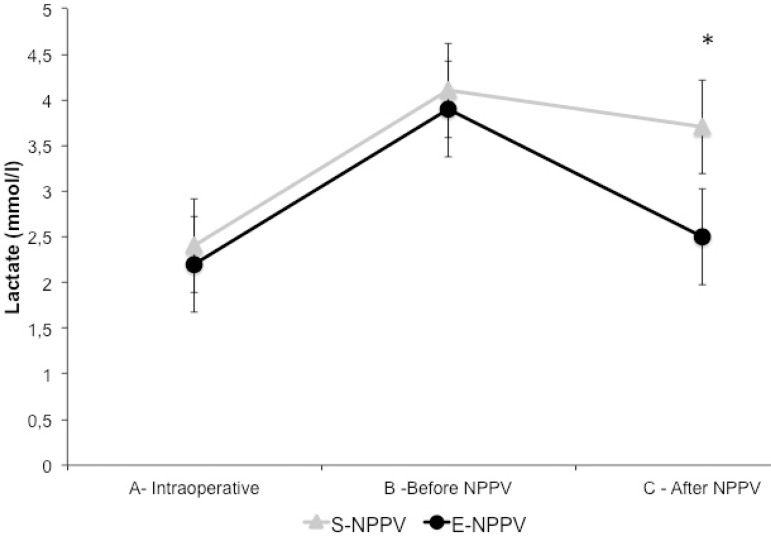
Sequential changes of blood lactate level before and after NPPV. *P<0.05. A=intraoperative (after anesthesia induction and invasive mechanical ventilation); B=before NPPV (spontaneous ventilation); C=after NPPV (immediately after NPPV protocol); E-NPPV=extended-time noninvasive positive pressure ventilation; S-NPPV=short-time noninvasive positive pressure ventilation

**Fig. 3:**
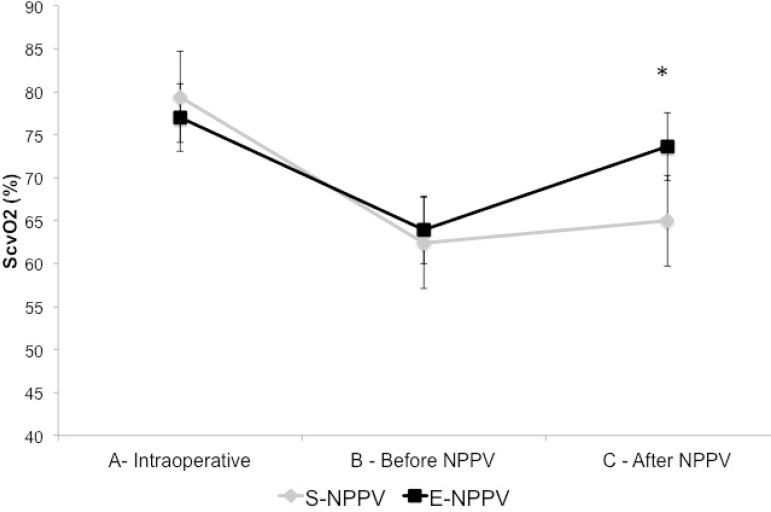
Sequential changes of ScvO_2_ before and after NPPV. *P<0.05. A=intraoperative (after anesthesia induction and invasive mechanical ventilation); B=before NPPV (spontaneous ventilation); C=after NPPV (immediately after NPPV protocol); E-NPPV=extended-time noninvasive positive pressure ventilation; S-NPPV=short-time noninvasive positive pressure ventilation; ScvO_2_=central venous oxygen saturation

After NPPV, a significant drop in blood lactate levels and an improvement in ScvO_2_ values were observed in the E-NPPV group when compared with S-NPPV group (*P*<0.05) (Figures 2 and 3, respectively).

In patients with blood lactate levels above 3 mmol/l (considering baseline values as 100%) during the IPO period, the use of E-NPPV lead to a 30.3% reduction in blood lactate levels. Conversely, the S-NPPV group demonstrated a 15.6% decline in blood lactate levels during the IPO period. In patients with ScvO_2_ values below 65% during the IPO period (considering baseline values as 100%), E-NPPV was able to increase in 23.3% the ScvO_2_ values, while in the S-NPPV group a 1.2% further decline was seen.

**Fig. 4:**
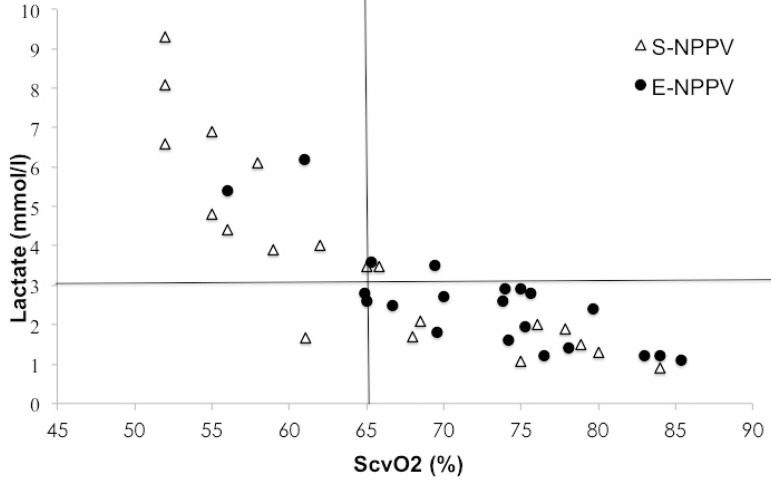
Patients' distribution according to cut-off values of 65% (ScvO_2_) and 3 mmol/L (lactate) after NPPV protocol. E-NPPV=extended-time noninvasive positive pressure ventilation; S-NPPV=short-time noninvasive positive pressure ventilation; ScvO_2_=central venous oxygen saturation

In Figure 4, the distribution of both groups was graphically analyzed regarding to blood lactate levels and ScvO_2_ values. Nine (45%) S-NPPV patients and two (9.5%) E-NPPV patients had blood lactate peak above 3 mmol/l and ScvO_2_ <65% (upper left quadrant). In the lower right quadrant, 17 (80.9%) E-NPPV patients and eight (40%) S-NPPV patients showed peak blood lactate levels below 3 mmol/l and ScvO_2_ >65%.

A significant impairment in FVC and FEV_1_ until POD5 was observed in both groups in comparison with the preoperative data (*P*<0.01). However, the E-NPPV group presented with higher FVC and FEV_1_ values on POD1, 3 and 5 than the S-NPPV group. Significant differences were found in FEV_1_ on POD1 between these groups. FVC and FEV_1_ were significantly different between S-NPPV and E-NPPV groups on POD5 (Table 2).

**Table 2:** Pulmonary function test values on postoperative days (POD) 1, 3, and 5, in percentage of preoperative values.

Variables	S-NPPV Group (n=20)		E-NPPV Group (n=21)	
	FVC (%)	FEV_1_ (%)	FVC (%)	FEV_1_ (%)
POD1	43.34±13.8	45.32±15.7	54.34±24.5*	55.84±12.4*
POD3	47.34±19.9	49.91±17.5	65.56±17.4*	72.38±15.1*
POD5	67.75±16.3	58.04±12.0	79.87±18.5*	80.45±16.3*

Data are shown as mean ± standard deviation. FVC and FEV1 are expressed in percentage considering 100% preoperative baseline value.

* *P*<0.05 for comparison between groups.

E-NPPV=extended-time noninvasive positive pressure ventilation; FEV_1_=forced expiratory volume in 1 second; FVC=forced vital capacity; S-NPPV=short-time noninvasive positive pressure ventilation

Hospital stay after CABG was significantly shorter in the E-NPPV group than in the S-NPPV group (*P*<0.05). Moreover, the incidence of respiratory events on POD5 was greater in the S-NPPV group than in the E-NPPV group (Table 3).

**Table 3:** Postoperative clinical variables.

Variables	S-NPPV Group (n=20)	E-NPPV Group (n=21)
Atelectasis % (n)	26.3 (5)	4.7 (1)*
Pleural effusion % (n)	15.7 (3)	9.5 (2)
Pneumonia % (n)	15.7 (3)	4.7 (1)*
Mechanical ventilation time (h)	12.1±1.8	11.2±1.1
Postoperative hospital stay (days)	8.1±2.1	6.9±1.3*

Data are shown as mean ± standard deviation. Comparison between the groups

* *P*<0.05. E-NPPV=extended-time noninvasive positive pressure ventilation; S-NPPV=short-time noninvasive positive pressure ventilation

## DISCUSSION

Prophylactic E-NPPV intervention demonstrated a positive impact on tissue perfusion, preservation of pulmonary function, and clinical outcomes compared to S-NPPV in the early postoperative period after CABG in this group of patients with preserved cardiac function. To our knowledge, this is the first study comparing the aforementioned outcomes between E-NPPV and S-NPPV after a major cardiac surgery.

A previous investigation^[^^11^^]^ demonstrated that the prophylactic use of E-NPPV is able to promote a better improvement in arterial oxygenation and some clinical outcomes than the S-NPPV. However, the above-mentioned study did not evaluate the effects of NPPV on tissue perfusion. Another study^[^^7^^]^ suggested that NPPV could be considered an effective and safe therapy to minimize dyspnea, improve tissue perfusion, and decrease arrhythmia frequency, reintubation rate, length of ICU stay, and mortality in patients after cardiac surgery when compared to oxygen therapy alone. Therefore, the novel aspects of our study were the influence of E-NPPV and S-NPPV application on tissue perfusion, pulmonary function, and clinical outcomes in patients who underwent CABG.

Recently, an investigation documented that a significant impairment in tissue perfusion (*i.e*. elevated lactate level and low ScvO_2_) could delay operation recovery, increase the risk of complications, and lead to a longer postoperative hospital stay, contributing to the risk of morbidity and mortality following CABG^[^^1^^-^^3^^]^. As a result, special interest has been given to strategies to improve tissue perfusion^[^^12^^]^.

In agreement with other studies^[^^1^^]^, our data revealed a significant increase in blood lactate levels and impairment in ScvO_2_ during the IPO period in comparison with preoperative values. According to Ranucci et al.^[^^13^^]^, ScvO_2_ and blood lactate level used in combination could be a clinical tool to help discern if an elevated lactate level is due to hypoperfusion or other mechanisms. In our study, a strong negative correlation supported the relationship between a ScvO_2_ drop below 65% and an increase in blood lactate levels. For this reason, we assume that blood lactate levels found in the current study were associated with impairment in tissue perfusion.

After NPPV application, there were a significant drop in blood lactate levels and a significant improvement in ScvO_2_ values in the E-NPPV group when compared with the S-NPPV group. Based on these findings, we believe that NPPV was the interventional mechanism for improvement in tissue perfusion in patients undergoing CABG, in particular E-NPPV.

In general, some potential mechanisms may explain our findings. Firstly, we believe that NPPV promoted a beneficial effect on cardiac function. After on-pump CABG, early reperfusion precipitates a period of relative hemodynamic instability in which small and rapid changes in ventricular loading, myocardial perfusion pressure, and endogenous inotropic state can change global ventricular performance considerably^[^^14^^]^. In addition, the early reperfusion period is characterized by a high incidence of regional or global ventricular dysfunction. Even in patients with a preoperative preserved ventricular function, a significant ejection fraction reduction could be noted in the first four hours after surgery^[^^15^^]^. Previous research has shown that NPPV application in patients with cardiac dysfunction could increase cardiac index, systemic oxygen delivery, and oxygen consumption^[^^16^^-^^19^^]^. Despite the fact that patients in the present study had preserved cardiac function, we believe that NPPV could have prevented acute cardiac alterations related to the operation. Secondly, studies have shown experimentally that atelectasis causes a significant increase in right ventricular afterload, thereby affecting left ventricular performance. This effect of atelectasis on right ventricular afterload during mechanical ventilation could be explained by two mechanisms: overdistention in aerated lung areas and local hypoxic pulmonary vasoconstriction in non-aerated lung areas^[^^20^^,^^21^^]^. In the current study, the E-NPPV group demonstrated a lower prevalence of atelectasis than the S-NPPV group. We speculate that E-NPPV reduced the risk of atelectasis and subsequently right ventricular stress by a reduction in hypoxic pulmonary vasoconstriction.

Zarbock et al.^[^^11^^]^ demonstrated that prophylactic NPPV application at least six hours after the operation increased pulmonary oxygen transfer, reduced pulmonary complications, and also decreased ICU readmission rates following elective cardiac surgery. Similar results were found in our study. The use of E-NPPV was associated with a significantly better preservation of pulmonary function than the S-NPPV.

A greater degree of pulmonary dysfunction increases the risk of pulmonary complications during the CABG postoperative course, which may result in longer postoperative hospital stay and increased mortality^[^^4^^-^^6^^]^. These results are in agreement with our findings; the S-NPPV group presented with a higher pulmonary function impairment which was associated with a significantly greater intubation time, occurrence of atelectasis, and pneumonia until POD5, and longer hospital stay than the E-NPPV group.

We were able to show that E-NPPV application significantly preserved pulmonary function and reduced respiratory events in patients deemed to be at low surgical risk. The literature had demonstrated that high-risk patients could potentially have more benefits with NPPV application^[^^7^^]^. Therefore, we speculate that the benefit of E-NPPV in high-risk patients following cardiac surgery could be even more pronounced, which may also lead to a more profound reduction in length of hospital stay.

### Limitations

Our study has some limitations that should be highlighted. The duration (one hour, twice a day) of NPPV after ICU discharge in the E-NPPV group may not have been enough to promote a faster return of the pulmonary function to baseline values. However, the E-NPPV protocol was able to promote a better preservation of pulmonary function and to prevent postoperative complications when compared to the S-NPPV.

## CONCLUSION

Prophylactic E-NPPV administered in the early postoperative period of CABG resulted in greater improvements in tissue perfusion, pulmonary function, and clinical outcomes than S-NPPV in patients with preserved left ventricular function. These findings hold clinical relevance and should be considered when developing the care plan for individuals undergoing a major cardiac surgery.

**Table:** 

Authors' roles & responsibilities	
MLSN	Concept, design, acquisition, analysis and interpretation of data, critical review of the study; final approval of the manuscript version to be published
DWB	Interpretation of data, and critical review of the study; final approval of the manuscript version to be published
YGL	Acquisition of data, final approval of the manuscript version to be published
FSP	Acquisition of data, final approval of the manuscript version to be published
RA	Critical review of the study, final approval of the manuscript version to be published
PRLL	Acquisition of data, final approval of the manuscript version to be published
GF	Acquisition of data, final approval of the manuscript version to be published
AMCS	Analysis and interpretation of data; final approval of the manuscript version to be published
NOM	Interpretation of data, critical review of the study; final approval of the manuscript version to be published
NH	Critical review of the study, final approval of the manuscript version to be published
SG	Concept, design, analysis and interpretation of data, critical review of the study; final approval of the manuscript version to be published
WJG	Concept, design, analysis and interpretation of data, critical review of the study; final approval of the manuscript version to be published
